# Supplementary material for: The fishing and natural mortality of large, piscivorous Bull Trout and Rainbow Trout in Kootenay Lake, British Columbia (2008–2013)
Source: PeerJ. 2017 Jan 10;5:e2874. doi: 10.7717/peerj.2874 (PMC5228508; doi:10.7717/peerj.2874)
Supplement: Article S1 [file peerj-05-2874-s001.pdf]

To replicate the analyses

1. Install R Version (>= 3.3.1) from https://cran.r-project.org.
2. Install JAGS (>= 4.2.0) from http://sourceforge.net/projects/mcmc-jags/files/ to the default location.
3. If using Windows install Rtools (>= 3.3.1) from https://cran.r-project.org/bin/windows/Rtools/ choosing the default options.
4. Cut and paste the following code into the R console to quickly generate non-convergent results.

```
install.packages("devtools") # if not already installed
devtools::install_github("poissonconsulting/klexr")

library(klexr)
replicate_results()
```

5. Open the `results` folder in the working directory to view the plots which are saved as png files.
6. For more information type `?replicate_results`.
