# Supplementary figures and images for: The fishing and natural mortality of large, piscivorous Bull Trout and Rainbow Trout in Kootenay Lake, British Columbia (2008–2013)
Source: PeerJ. 2017 Jan 10;5:e2874. doi: 10.7717/peerj.2874 (PMC5228508; doi:10.7717/peerj.2874)

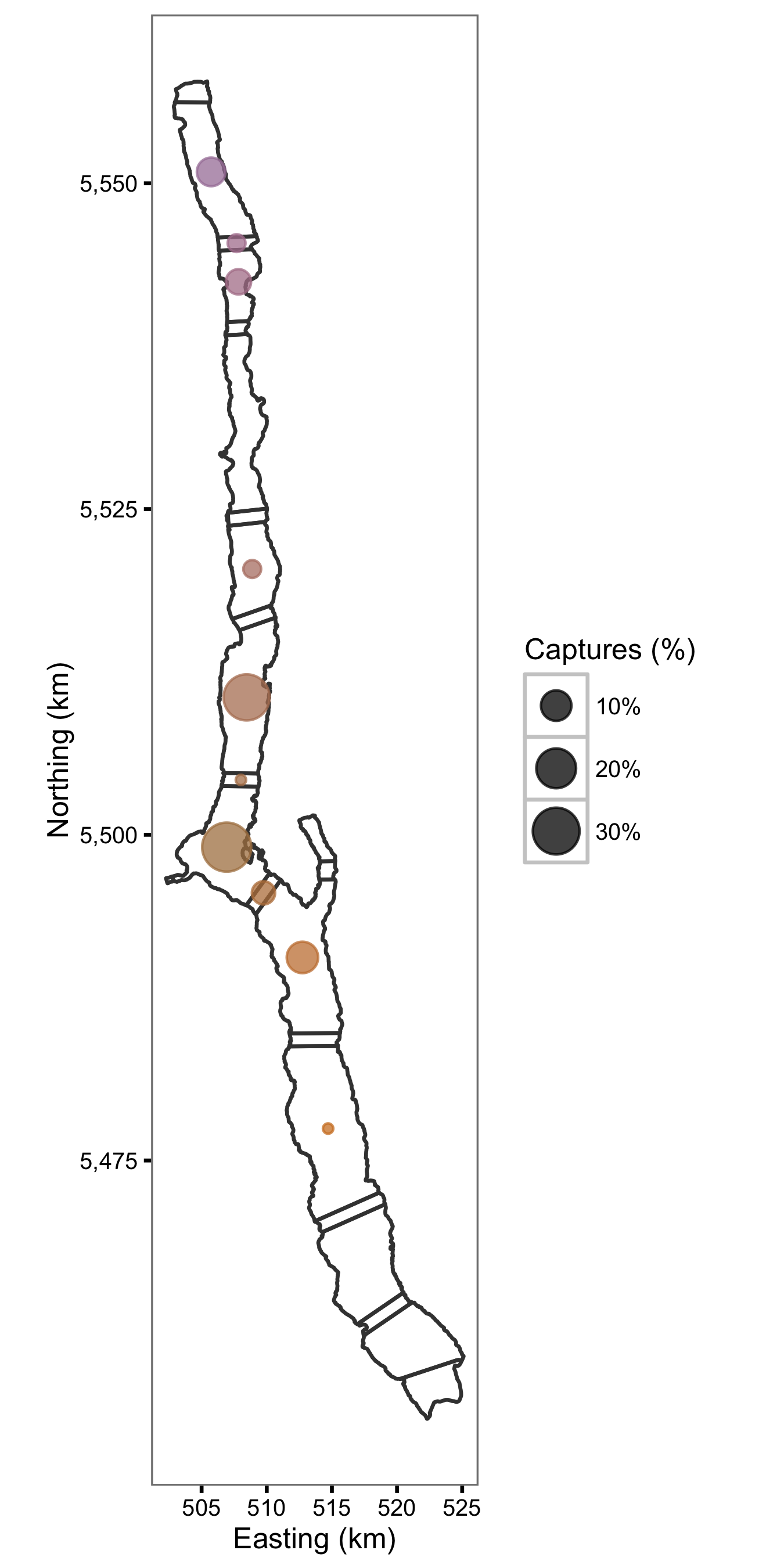

Supplement: Figure S1 — Spatial information licensed under the Open Government License of British Columbia. [file peerj-05-2874-s002.png]

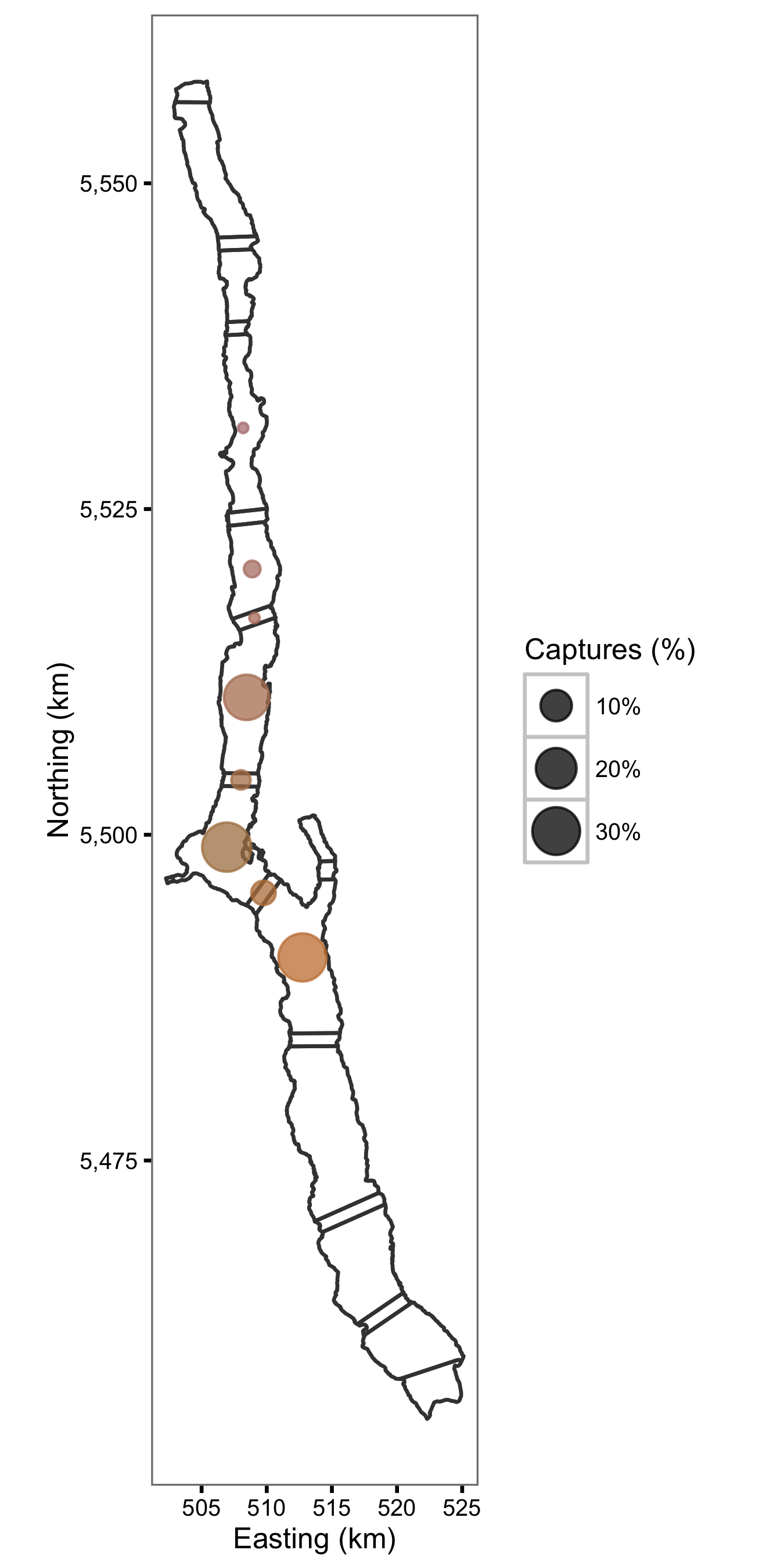

Supplement: Figure S2 — Spatial information licensed under the Open Government License of British Columbia. [file peerj-05-2874-s003.png]

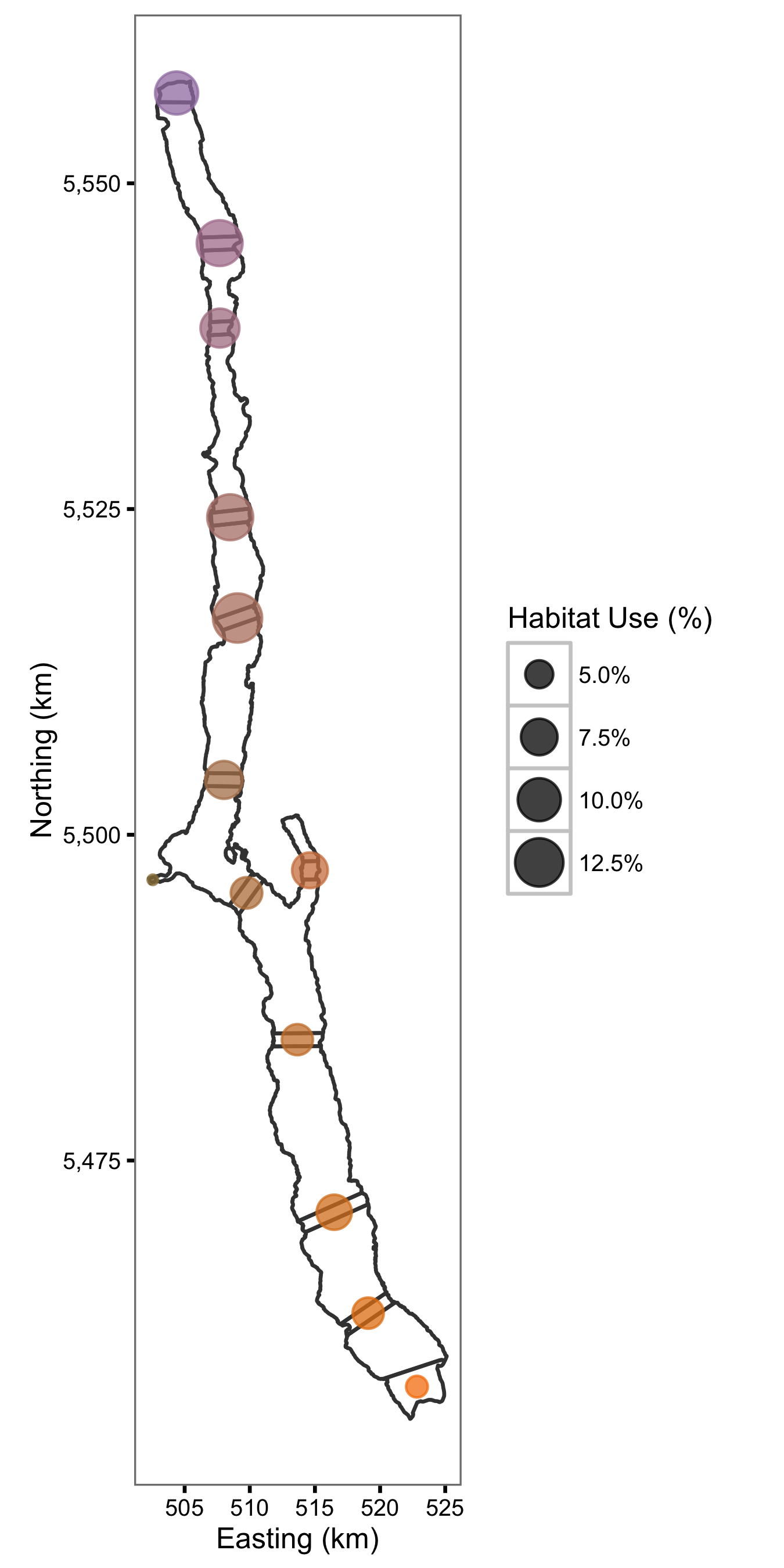

Supplement: Figure S3 — Spatial information licensed under the Open Government License of British Columbia. [file peerj-05-2874-s004.png]

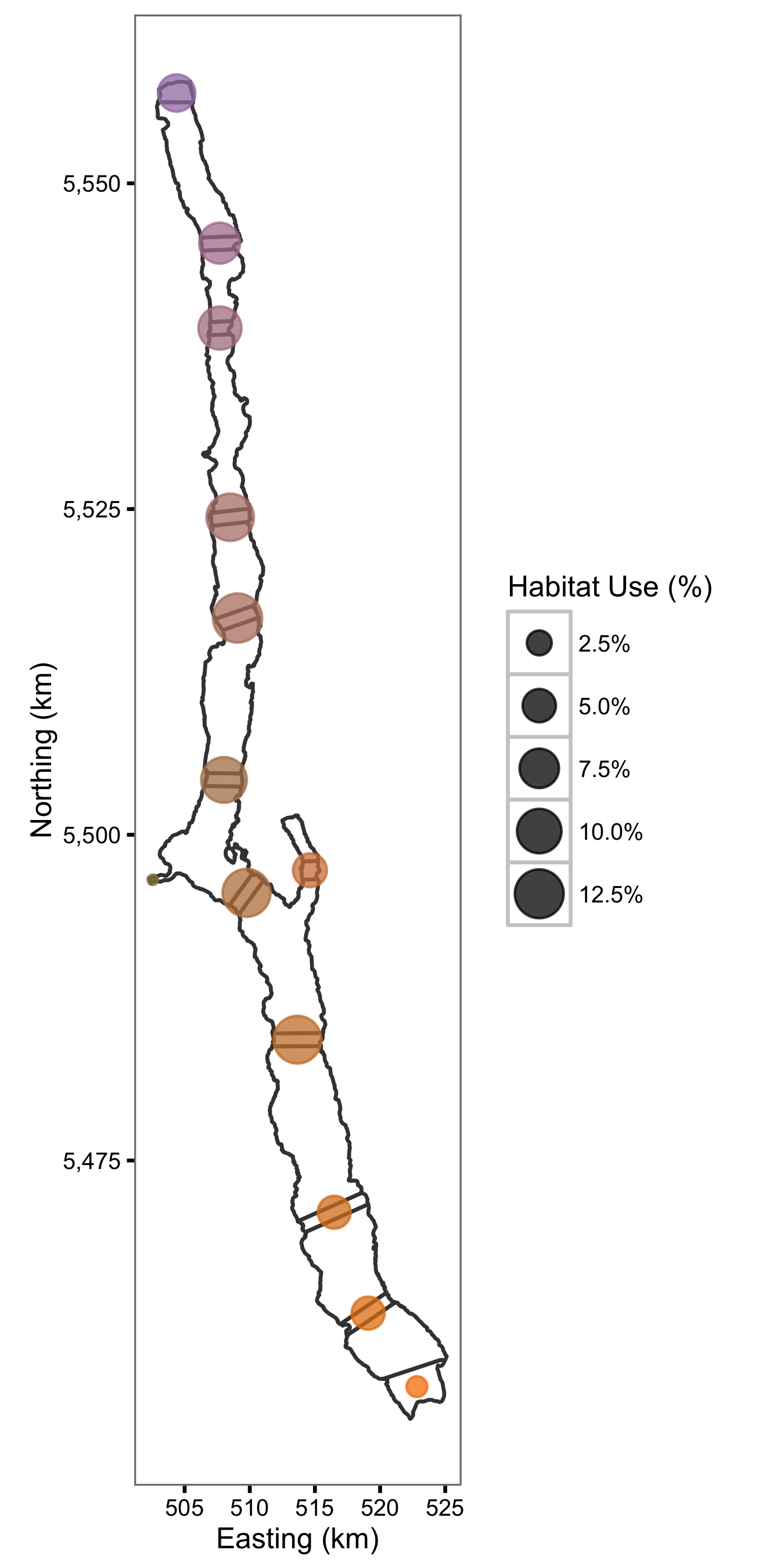

Supplement: Figure S4 — Spatial information licensed under the Open Government License of British Columbia. [file peerj-05-2874-s005.png]

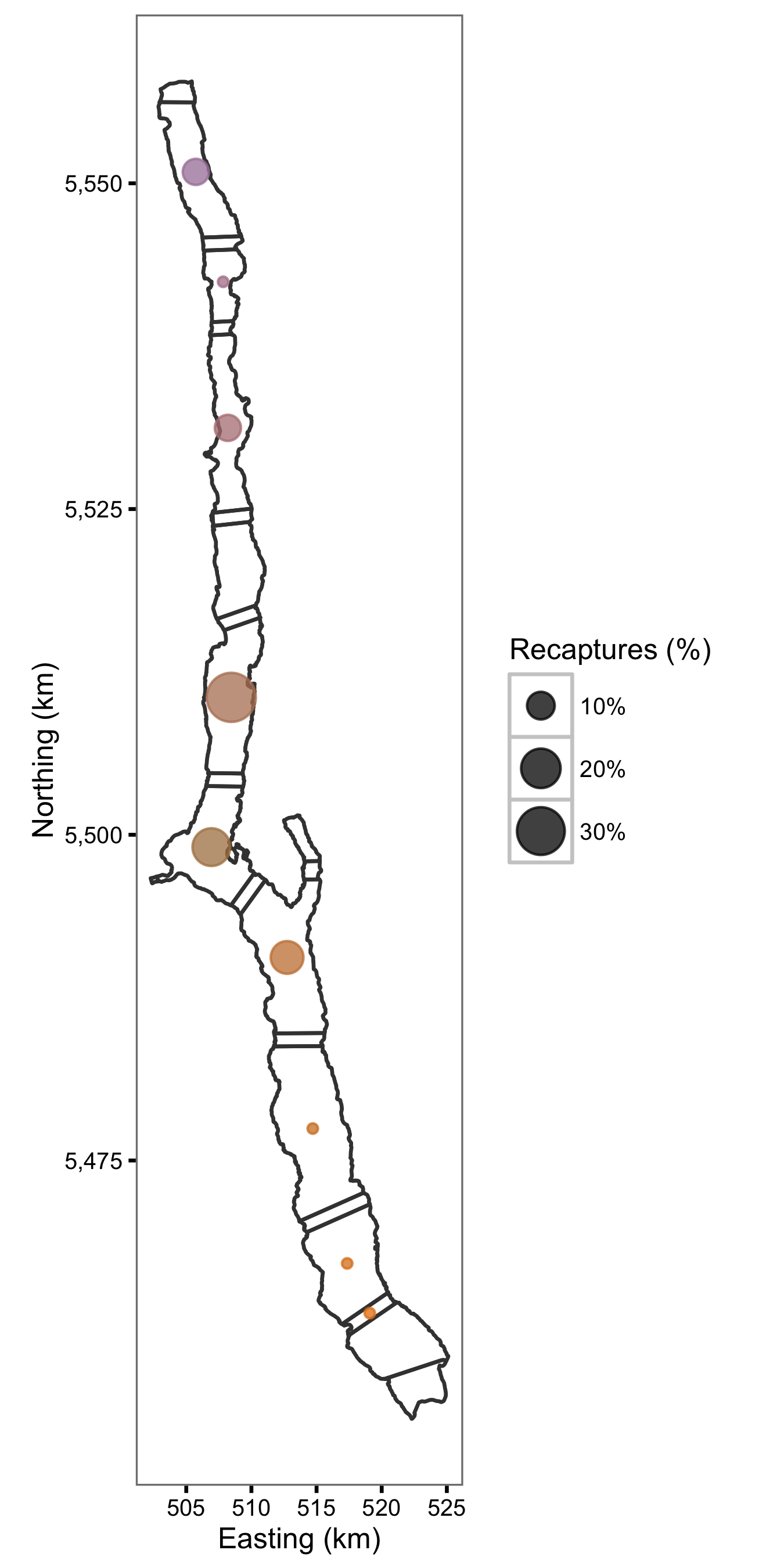

Supplement: Figure S5 — Spatial information licensed under the Open Government License of British Columbia. [file peerj-05-2874-s006.png]

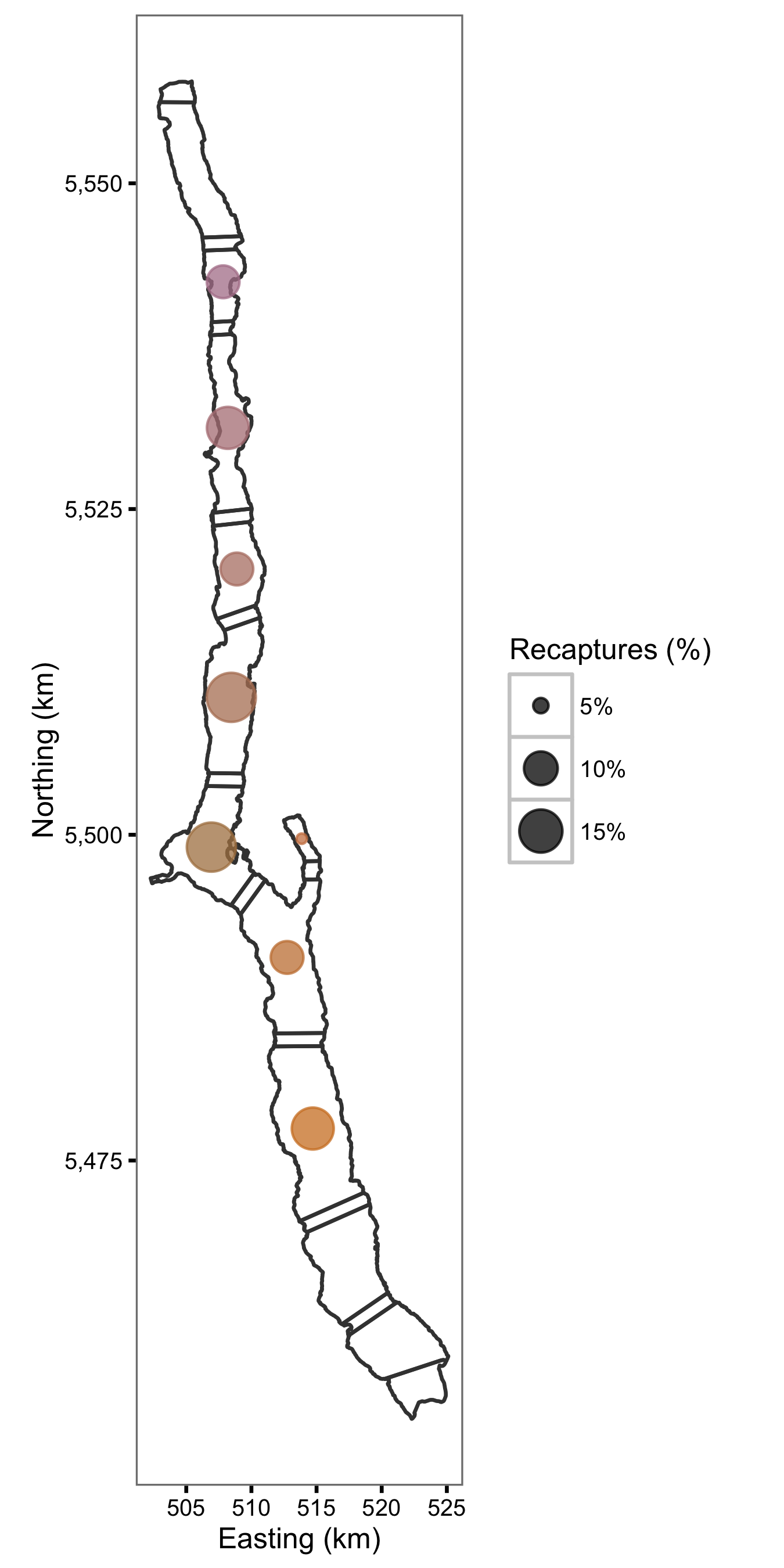

Supplement: Figure S6 — Spatial information licensed under the Open Government License of British Columbia. [file peerj-05-2874-s007.png]
